# Phytochemicals and Irisin as Multi-Target Regulators of Adipose Tissue Browning and Metabolic Reprogramming: Synergies with GLP-1 Pathways

**DOI:** 10.3390/antiox15091143

**Published:** 2026-09-09

**Authors:** Nuriye Nuray Ulusu

**Affiliations:** 1 School of Medicine, Department of Medical Biochemistry, Koç University, Sariyer, 34450 Istanbul, Turkey; nulusu@ku.edu.tr; 2 Koç University Research Center for Translational Medicine (KUTTAM), Sariyer, 34450 Istanbul, Turkey

**Keywords:** obesity, phytochemicals, berberine, resveratrol, catechins, capsaicin, thymoquinone, phycocyanin, irisin, metabolic regulation, adipose tissue browning, thermogenesis, mitochondrial biogenesis, inflammation, AMPK signaling, brain–gut–adipose tissue–liver axis

## Abstract

**Background:** Obesity is a multifaceted metabolic disorder characterized by systemic disturbances, particularly impaired energy homeostasis, chronic low-grade inflammation, and mitochondrial dysfunction across the brain, gut, adipose tissue, and liver axes. **Objectives:** This review aims to examine the metabolic properties and molecular mechanisms of six key phytochemicals (berberine, resveratrol, catechins, capsaicin, thymoquinone, and phycocyanin) and the exercise-induced myokine irisin, and their roles in mitochondrial signaling and metabolic reprogramming. **Sources of Evidence:** A comprehensive literature search was conducted across major electronic databases, including PubMed, Web of Science, and Scopus, to identify relevant mechanistic, in vivo, and in vitro studies. **Results:** Both the selected phytochemicals and irisin act as multi-target regulators that modulate key signaling pathways, including AMPK, PI3K/Akt/mTOR, SIRT1, Nrf2, and PPARγ. These phytochemicals and irisin can drive cell- and tissue-specific metabolic reprogramming, promoting the browning of white adipocytes, suppressing de novo lipogenesis in hepatocytes, and enhancing fatty acid oxidation in skeletal myocytes. This synergistic metabolic reprogramming enhances thermogenesis and increases energy expenditure. **Conclusions:** Co-targeting redox signaling and metabolic pathways via phytochemicals and irisin offers a powerful strategy against obesity. This integrative framework restores multi-organ homeostasis, laying the groundwork for targeted metabolic therapies.

## 1. Introduction

Obesity, overweight, and metabolic syndrome represent major health problems worldwide. These conditions are closely related to a range of metabolic disorders and genetic, environmental, behavioral, socioeconomic, dietary, and lifestyle factors [1,2,3,4]. They increase the risk of insulin resistance, diabetes, cancer, and double the risk of coronary heart disease, steatotic liver disease, polycystic ovary syndrome, and other obesity-associated comorbidities [5,6]. Obesity is not a simple disease directly correlated with the energy surplus. It is a chronic multifactorial disease that covers a group of metabolic reprogramming, biochemical, and physiological dysfunctions [7], including mitochondrial dysfunction [8], inter-organ communication failure [9], chronic low-grade inflammation [10], neuroendocrine dysregulation [11], gut microbiota dysbiosis [12], redox imbalance [13], and impaired thermogenesis [14].

Certain bioactive plant metabolites have beneficial effects on human glucose and lipid metabolism, modulate cellular functions and signaling pathways to reduce oxidative stress, enhance mitochondrial function, and support healthy metabolic homeostasis [15], and slow the aging process [16]. The term ‘metabolic reprogramming’ is defined as tissue- and cell-specific metabolic remodeling and mitochondrial adaptations; specifically, the transdifferentiation of energy-storing white adipocytes into thermogenic beige adipocytes, the suppression of de novo lipogenesis in hepatocytes, and the upregulation of fatty acid β-oxidation and glucose catabolism in skeletal myocytes [17,18]. At the subcellular level, metabolic reprogramming is tightly regulated by mitochondrial dynamics. The mitochondrial-derived peptide MOTS-c counters insulin resistance not only by triggering mitochondrial biogenesis (via TFAM, COX4, and NRF1) but also by promoting mitochondrial fusion (mitofusion) through OPA1 and mitofusin 2 (MFN2). Crucially, suppressing these fusion GTPases, either via TNF-α or MFN2 knockdown, eliminates MOTS-c-induced GLUT4 translocation and glucose uptake. This establishes a direct biochemical link between mitochondrial fusion dynamics and insulin sensitivity [19]. These phytochemicals lower blood glucose levels, protect against diabetes, obesity, hyperlipidemia, and atherosclerosis, and reduce the risk of metabolic disorders [20]. Various phytopharmaceutical compounds affect uncoupling protein 1 (UCP1) and trigger UCP1-dependent or UCP1-independent thermogenesis [21]. UCP1, also referred to as thermogenin, is a key mediator of mitochondrial uncoupling in brown adipose tissue [22]. WAT browning is a metabolic remodeling process in which white adipocytes acquire beige-like features, including increased mitochondrial biogenesis, elevated UCP1 expression, and enhanced thermogenic capacity, thereby promoting energy expenditure [23,24]. Berberine has anti-adipogenic effects and increases lipolytic activity [25], activates energy-related pathways, promotes the release of energy as heat rather than chemical energy, modulates lipid metabolism, and induces white adipose tissue (WAT) to convert to brown adipose tissue (BAT) [25,26]. Resveratrol induces mitochondrial biogenesis and AMPK activation, and increases NAD+ levels, thereby activating SIRT1, which is essential for resveratrol-mediated regulation of energy homeostasis and is linked to AMPK activation and potential anti-obesity benefits [27]. Resveratrol promotes WAT browning, a key mechanism for burning excess fat [28]. Catechins, specifically green tea catechins, rich in epigallocatechin gallate (EGCG), have thermogenic effects that improve fat oxidation [29]. Capsaicin modulates adipokine gene expression, has a thermogenic effect, reduces appetite, and suppresses inflammation [30]. Nigella sativa contains thymoquinone and a variety of bioactive compounds that can be used to treat chronic inflammatory diseases, such as dyslipidemia, hypertension, and type 2 diabetes mellitus [31]. Spirulina contains phycocyanin, which has beneficial effects on metabolic syndrome, reducing obesity and body mass index (BMI) [32]. Irisin is a 112-amino-acid hormone released in response to exercise in mice and humans, and it actively regulates metabolic reprogramming [33]. Irisin affects WAT, promotes brown adipocyte formation, and increases energy expenditure [34]. Within this systemic network, phytochemical-mediated gut microbiota optimization and anti-inflammatory remodeling (such as the M1-to-M2 macrophage transition) are not isolated events; they directly resolve tissue-level stress, thereby unlocking and amplifying the core thermogenic and browning potential of beige adipocytes. These pathways act through networks that complement or mirror GLP-1 receptor agonists [35,36].

Driven by a strong focus on metabolic health and the physiological challenges of weight management, this review addresses the need for a unified understanding of multi-organ crosstalk. This review aims to describe the effects of berberine, resveratrol, catechins, capsaicin, thymoquinone, and phycocyanin, together with the fundamental role of exercise-induced irisin, on the activation of key metabolic signaling pathways related to increased mitochondrial biogenesis, thermogenesis, fatty acid oxidation, insulin sensitivity, and decreased inflammation, oxidative stress, gut microbiota modulation, adipose tissue browning, and systemic metabolic homeostasis. Phytochemical-induced modulation and irisin-mediated signaling across the multi-systemic brain–gut–adipose tissue–liver axis provide anti-obesity strategies and enhance metabolic homeostasis, acting through networks that complement or mirror GLP-1 receptor agonists.

## 2. Methodology

### 2.1. Literature Search Strategy

A comprehensive and systematic literature search was conducted on 28 June 2026, which served as the date of the most recent search update. The primary screening and identification of evidence were conducted across major electronic databases, including PubMed/MEDLINE, Web of Science, Scopus, and Google Scholar, with dates of coverage spanning from 1 January 2000 to June 2026. The search strategy was designed to systematically capture peer-reviewed studies evaluating the molecular, cell-signaling, and biochemical mechanisms of phytochemicals in obesity and metabolic syndrome.

The following Medical Subject Headings (MeSH) terms and keywords, along with Boolean operators (AND/OR), were utilized to maximize search sensitivity:

(phytochemicals, name of the phytochemical, and UCP1 OR natural compounds OR polyphenols OR flavonoids OR berberine OR EGCG OR capsaicin)
AND (obesity OR metabolic syndrome OR adipose tissue OR browning OR thermogenesis)
AND (AMPK OR SIRT1 OR FAS OR “mitochondrial dysfunction” OR inflammation).

Additionally, the reference lists of retrieved reviews and key papers were manually cross-referenced to identify any further eligible studies that might have been missed during the primary electronic search. Direct contact with primary study authors was not initiated, as the electronic database indexing combined with manual reference tracking provided a highly saturated and sufficient body of evidence containing 322 curated sources.

### 2.2. Eligibility Criteria and Source Characteristics

To confirm a healthy, reliable, and scientifically rigorous synthesis of the evidence, specific eligibility criteria were strictly applied to the selected sources:

Publication Period and Rationale: The literature selection strategy heavily prioritized the most recent scientific breakthroughs, with most included sources published within the last three years (2023–2026). This specific focus ensures that the review reflects contemporary advancements in adipose tissue plasticity, omics technologies, and gut microbiota crosstalk. However, carefully selected foundational landmark studies from earlier years were intentionally included. The inclusion of these older sources is crucial to preserve mechanistic continuity and to properly cite the pioneering discoveries of foundational enzymatic and molecular pathways that underpin current obesity research.

Only peer-reviewed articles published in English were considered. This restriction ensures international reproducibility, standardization of complex biochemical, genetic, and pharmacological terminology, and minimizes the risk of translation or interpretation bias.

Eligible sources were strictly restricted to peer-reviewed original research articles (in vitro, in vivo, and clinical trials), systematic reviews, and meta-analyses. Gray literature, conference abstracts, academic dissertations, book reviews, and unreviewed preprints were excluded to guarantee that the synthesized data possesses the highest level of methodological rigor and scientific credibility.

### 2.3. Data Extraction and Synthesis

Following the initial electronic search, duplicate citations were removed. Titles and abstracts were screened independently to assess their alignment with the multi-target mechanisms of natural compounds in metabolic disorders. Data regarding the specific phytochemical classes, target signaling pathways (e.g., AMPK/SIRT1/PGC-1α), structural adaptations (white versus brown fat browning), and physiological outcomes were systematically extracted. The finalized evidence from the 321 curated references was synthesized into distinct thematic sections and structured summary tables to ensure analytical clarity.

## 3. The Role of Key Tissues in the Brain–Gut–Adipose Tissue–Liver Axis

### 3.1. Fundamental Molecular and Physiological Mechanisms of Obesity

Obesity is a complex, multifactorial progressive metabolic disorder that extends far beyond simple positive energy balance, including genetic, molecular, environmental, and behavioral interactions [37]. Pathophysiology of obesity involves systemic metabolic disorders, chronic inflammation, and metabolic disruption [38]. Obesity represents a whole-body network failure marked by disrupted communication across the neuroendocrine, adipose, hepatic, gut, and immune axes. This multi-organ breakdown fuels chronic metabolic inflammation (metainflammation) and premature cellular aging [39].

Energy balance is regulated by the brainstem and hypothalamus, whereas emotional eating engages the brain’s reward centers. These regions together control appetite and satiety [40]. Gut hormones like CCK, GLP-1, and PYY signal through the vagus nerve to reduce hunger, while the gut microbiome and neurotransmitters also help regulate satiety [41]. Targeting gut–adipose communication offers a promising avenue for developing personalized therapies against metabolic disorders [42].

Specifically, intestinal media reduce hepatic triglycerides and modulate key lipid regulators, such as resistin, FGF21, SREBP-1c, NRF2, Src, AMPK, SIRT1, and PGC1α, via GLP-1 signaling. Downstream, hepatic media reduce adipocyte lipid accumulation and upregulate browning markers such as UCP1, NOS, SIRT1, and STAT3, and highlight multi-tissue crosstalk as a target for plant-derived interventions [43].

#### 3.1.1. Neuroendocrine Dysregulation and Central Appetite Control

The hypothalamus, especially the arcuate nucleus (ARC), ventromedial (VMH), dorsomedial (DMH); paraventricular (PVN) nuclei, plays a critical role in metabolic signals and neuroendocrine dysregulation effects glucose homeostasis and central energy sensing [44]. Gut GLP-1, CCK, PYY, and ghrelin, along with pancreatic PP and adipocyte leptin, coordinate appetite along the brain–gut–adipose axis, though this homeostatic control is often overridden by environmental factors in obesity [45]. Overnutrition and hyperleptinemia impair JAK2-STAT3 signaling, reduce blood-brain barrier permeability, cause defective autophagy, endoplasmic reticulum stress, inflammation, decreased leptin receptor expression, and increased mTOR activity, which would cause leptin resistance [46]. Leptin plays an important role in obesity and insulin resistance [47].

#### 3.1.2. Adipose Tissue Remodeling and Local Inflammation

White adipose tissue is a key endocrine organ that maintains metabolic balance. In obesity, dynamic cellular changes, such as fibrosis, immune infiltration, and cell stress, lead to dysfunction, chronic inflammation, and insulin resistance [48]. In obesity, the reduction in adiponectin impairs its protective role, which normally suppresses pro-inflammatory cytokines and promotes M2 macrophages to prevent metabolic inflammation [49]. Beyond genetic and dietary drivers of adipocyte hypertrophy and hyperplasia, syndromic obesity models offer unique insights into the peripheral mechanisms regulating adipose tissue remodelling [50]. Dietary bioactive compounds induce white adipose tissue browning by activating crucial thermogenic signaling pathways [51].

#### 3.1.3. Lipotoxicity and Systemic Metabolic Complications

Chronic calorie excess and adiposity lead to ectopic fat accumulation across various tissues, ultimately impairing organelle function [52]. This lipid-driven adipose dysfunction triggers systemic lipotoxicity and altered multi-tissue signaling, ultimately exacerbating obesity-associated metabolic and cardiovascular pathologies in obesity [53]. Lipotoxicity promotes hepatic insulin resistance and NAFLD through PKC/JNK-1 pathway activation, mitochondrial dysfunction, and endoplasmic reticulum stress [54]. Ectopic fat deposition and altered lipid metabolism directly link non-alcoholic fatty liver disease (NAFLD) with non-alcoholic fatty pancreas disease (NAFPD), making the early diagnosis of NAFLD crucial for halting pancreatic disease progression [55].

#### 3.1.4. Gut–Liver–Adipose Axis and Microbiome Disruption

Disruptions in the gut microbiota reduce bacterial diversity and drive obesity by altering host metabolism and triggering inflammation, making microbiome restoration a potential therapeutic pathway for weight management [56].

Obesity is driven by intestinal barrier dysfunction and metabolic endotoxemia, where gut-derived lipopolysaccharides trigger chronic inflammation and systemic metabolic impairment along the gut–liver–adipose axis [57].

### 3.2. Molecular Targets of Phytochemicals in Axis Homeostasis

**Figure 1:**
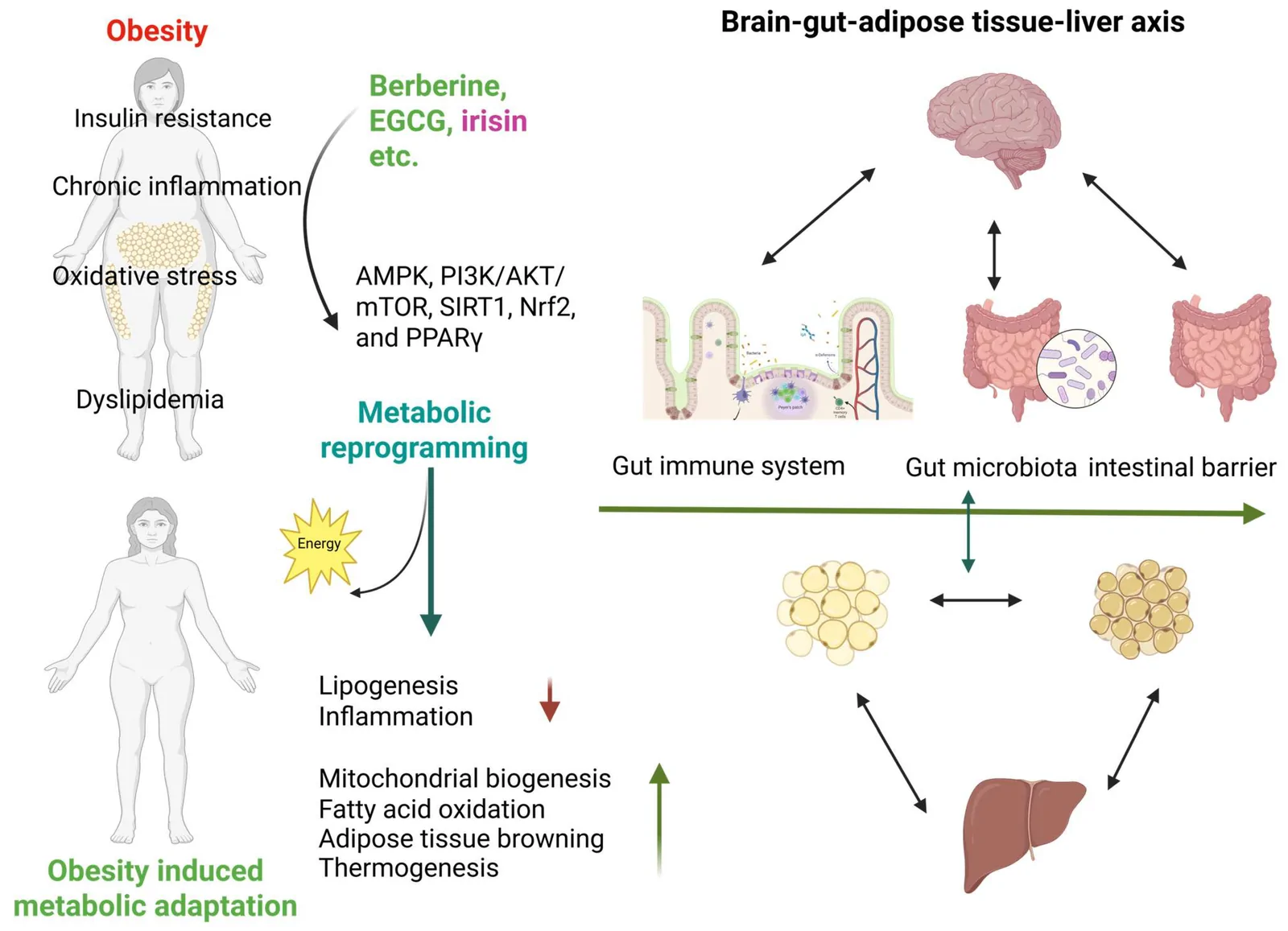
The brain–gut–adipose tissue–liver axis and the regulatory effects of phytochemicals on metabolic signaling pathways involved in obesity.

Phytochemicals have been shown to reduce obesity by regulating energy homeostasis by targeting metabolic pathways and substrates such as glucose and fatty acids [58]. Specifically, AMPK, PI3K/Akt/mTOR, SIRT1, Nrf2, and PPARγ are target pathways of phytochemicals that regulate energy balance and inflammatory responses at the transcriptional level. Activation of the AMPK/SIRT1/PGC-1α axis serves as a central regulator of cellular energy homeostasis, epigenetic modifications, and transcriptional programs [59]. Figure 1 summarizes the complex regulatory interactions within the brain–gut–adipose tissue–liver axis that maintain metabolic homeostasis.

These signaling pathways can regulate glucose and lipid catabolism, reduce inflammation, and enhance mitochondrial function, all of which are critically important for the metabolic homeostasis of insulin-sensitive tissues [60,61,62,63,64,65]. Obesity is associated with the gut–brain axis [66], the gut–liver axis [67], and communication among the brain, gut, and adipose axes [68].

#### 3.2.1. Brain Energy Sensing and Appetite

Cellular metabolism depends on the availability of substrates and the controlled activity of metabolic allosteric enzymes that maintain balance between food intake, energy requirements, and body weight regulation [69,70]. The brain regulates appetite, behavioral food intake, and energy metabolism via neuroendocrine signals involved in starvation responses, anorexia, obesity, and energy homeostasis [71,72]. Disrupted central and peripheral hormonal signaling impairs appetite control and contributes to the development of obesity [73]. Ghrelin is secreted from the stomach and increases food intake and secretion of growth hormone [74]. Oxyntomodulin, secreted from the intestine during digestion, increases acid secretion [75]. Anorexigenic hormones, cholecystokinin (CCK), pancreatic polypeptide (PP), peptide YY (PYY), and glucagon-like peptide 1 (GLP-1), released from the gastrointestinal system and various organs, decrease food intake, reduce acid, pancreatic secretions, and slow gastric mobility [73]. Furthermore, the rate of gastric emptying or gastric accommodation is among the motor functions of the stomach. The stomach sends signals to the brain through nerves, especially the vagus nerve, about how full the stomach is, which regulates appetite and meal size [76]. Food intake is under the control of several mechanisms, such as CD36/SR-B2, an important scavenger receptor that is expressed in enterocytes and the brain, which binds long-chain fatty acids and plays a significant role in the tongue–gut axis relation to appetite [77].

#### 3.2.2. The Gut Microbiota in Energy Homeostasis and Inter-Organ Crosstalk

The gut microbiota plays a key role in energy homeostasis and can increase energy expenditure and protect against obesity, affect inflammation, insulin resistance, and metabolic dysfunction by bidirectional crosstalk [78]. Gut microbiota modulates leptin expression and sensitivity in a diet-dependent manner, which affects body-weight regulation through epigenetic and metabolic mechanisms [79]. The gut immune system and barrier function, specifically permeability, are altered in obesity and play a critical role in neurodegenerative diseases and aging. This process is driven primarily by the gut microbiota [80,81]. Crosstalk between adipose tissue and gut microbiota is bidirectional. Adipose tissue affects microbiota by secreting leptin, adiponectin, fibroblast growth factor 21 (FGF21), apelin, and the gut microbiota affects adipose tissue by secreting short-chain fatty acids (SCFAs), bile acids, LPS, and the endocannabinoid (eCB) system [78]. The gut microbiota regulates physiological processes, such as appetite and energy balance, taste receptor signaling, and dysbiosis may alter perception and food taste [82]. Gut microbiota-derived SCFAs, such as acetate, propionate, and butyrate, play a key role in gastrointestinal dysfunction, obesity, and type 2 diabetes mellitus [83].

Obesity causes intestinal barrier damage because of inflammatory and oxidative processes [84].

The gut–brain axis (GBA) is bidirectionally modulated by gut microbiota through neuronal, endocrine, immune, and humoral pathways [85]. This bidirectional communication highlights that gut microbiota optimization is not an isolated gastrointestinal event, but a fundamental driver of systemic energy expenditure [86]. Specifically, phytochemical-mediated enrichment of beneficial bacterial strains increases the production of short-chain fatty acids (SCFAs) like butyrate and acetate [87].

These SCFAs cross the gut barrier to stimulate sympathetic nervous system outflow and induce the secretion of glucagon-like peptide-1 (GLP-1), which directly upregulates UCP1 expression in adipose tissue [88]. Phytochemicals break this pathological cycle through a dual-action mechanism: they promote an anti-inflammatory M2 macrophage phenotype in adipose tissue while simultaneously optimizing gut microbial communities [36]. This reduction in systemic inflammation facilitates the polarization of adipose tissue macrophages from a pro-inflammatory M1 to an anti-inflammatory M2 phenotype, creating the optimal microenvironmental conditions required for irisin and catecholamines to drive efficient white-to-beige adipocyte transdifferentiation [35].

#### 3.2.3. Adipose Tissue Dysregulation, Inflammation, and Thermogenic Plasticity

Adipose tissue (AT) dysfunction plays a key role in the pathophysiological process in obesity-related disorders [89]. Metabolic dysregulation of hormones, such as leptin, which is secreted from WAT and regulates food intake, body mass, proinflammatory immune response, and lipolysis [90]. Adipose tissue has two sites: the passive site is for energy storage. The active site regulates the whole body’s physiology, maintains energy levels, body temperature, insulin sensitivity, and immune responses [91]. Adipose tissue and adipocytes are highly plastic in size and number, and, under certain conditions, adipocytes can interconvert from white to BAT. This interconversion is due to mitochondrial biogenesis, which increases the thermogenic property [92]. Adipose tissue is an extraordinarily flexible and heterogeneous organ, and it undergoes significant phenotypic, metabolic, and structural adaptations [91]. In obesity, dynamic alterations in adipose tissue are characterized by pathological changes such as fibrosis, immune cell infiltration, endoplasmic reticulum stress, ectopic lipid accumulation, low-grade inflammation, and insulin resistance [93]. All these cellular alterations may cause metabolic disturbances, organ damage [94], and muscle ageing [95]. Located in the inner mitochondrial membrane, UCP1 disrupts the proton gradient, uncoupling respiration from ATP synthesis to generate heat. Thus, it serves as a promising therapeutic target for metabolic regulation [96].

Each of these factors is interconnected and creates a synergistic network of metabolic regulation, acting via direct binding to nuclear receptors or kinases that gate the feedback loop, or through nutrient-sensing pathways such as SIRT1 and AMPK [97]. Every molecule that enters the cell may have multi-node functions and change metabolism, even the transition of adipose tissue from white to beige, then to brown phenotypes, which reflects increased mitochondrial biogenesis [98].

#### 3.2.4. Irisin: An Exercise-Induced Myokine Orchestrating Tissue Browning and Systemic Metabolism

Irisin is a novel myokine and has a key role in metabolic regulation and programming [99]. The exercise-induced myokine is secreted by skeletal muscle cells. It is also secreted from adipose tissue, earning its name adipokine. Irisin exerts multi-spectrum functions [99,100,101], including osteogenesis [102]. Irisin acts as a pivotal biochemical link connecting the benefits of physical activity to the underlying mechanisms of disease pathophysiology [103]. As a cleavage product of the transmembrane protein FNDC5, irisin acts as a critical link between physical exercise and metabolic homeostasis, driving energy expenditure through the thermogenic activation of adipose tissue [104]. While traditionally recognized as an exercise-induced metabolic hormone, irisin has recently been reported to possess robust antioxidative properties capable of suppressing oxidative stress [105]. Irisin plays a key role in metabolism and is associated with metabolic syndrome [106]. Irisin exhibits anti-inflammatory effects by inhibiting NF-κB signalling, enhances brain-derived neurotrophic factor (BDNF) signalling, has beneficial effects on insulin resistance, and decreases amyloid-β toxicity and oxidative stress [107]. Irisin is at the crossroads of interorgan communication and is found in muscle and adipose tissue, and then in bone, heart, liver, and brain [108]. Irisin stimulates adipose tissue browning via PGC-1α, peroxisome proliferator, PPAR γ, and other downstream mediators to induce UCP-1 during exercise [109]. Irisin reduces pro-inflammatory cytokines and increases anti-inflammatory cytokines [110]. AMPK and mTOR are the initial steps of autophagy processes, and irisin is an important regulator of autophagy by exercise [111]. The primary function of irisin is upregulation of UCP1, which leads to mitochondrial biogenesis and transdifferentiation of WAT to beige or brown type [112]. Irisin regulates hepatic glucose metabolism by activating AMPK and PI3K/Akt by affecting the activities of glycogen storage and gluconeogenesis enzymes, glucose-6-phosphatase and phosphoenolpyruvate carboxykinase [113]. Irisin inhibits lipogenesis [114] and has protective effects on hepatic steatosis [115].

#### 3.2.5. Phytochemical Synergy with the Irisin/GLP-1 Axis in Adipose Browning

Irisin and GLP-1 activate parallel intracellular signaling cascades that enhance glucose-stimulated insulin secretion (GSIS), contributing significantly to systemic energy homeostasis, glycemic control, and weight management [116]. In human white adipocytes, irisin exhibits dual metabolic effects by simultaneously promoting thermogenic browning and modulating adipogenesis. Specifically, treatment of human subcutaneous white adipose tissue (scWAT) with irisin increases UCP1 expression through the activation of ERK and p38 MAPK signaling pathways [117]. Furthermore, irisin activates the AMPK/PGC-1α/PPARγ axis to stimulate mitochondrial biogenesis and thermogenic fat burning, while concurrently reducing inflammatory cytokine expression by inhibiting NF-κB nuclear translocation [118,119].

Crucially, selected phytochemicals directly synergize with this irisin-driven metabolic network [120,121]. Specifically, berberine and thymoquinone stimulate the upstream AMPK/PGC-1α pathway to upregulate endogenous FNDC5/irisin expression [121,122,123]. Meanwhile, capsaicin activates TRPV1 signaling to amplify UCP1-driven thermogenesis [124]. Furthermore, phytochemicals such as curcumin, resveratrol, and EGCG suppress NF-κB-mediated chronic inflammation and modulate PI3K/Akt/PPARγ signaling, creating a permissive microenvironment that potentiates irisin-driven white-to-beige adipocyte transdifferentiation [101,125,126,127,128].

#### 3.2.6. Hepatic Lipid Trafficking, Insulin Resistance, and Steatotic Liver Diseases

The liver is the center of carbohydrate, lipid, and amino acid metabolism that integrates signals from the gastrointestinal tract and adipose tissue [129]. Acetate and ethanol from the gut, and non-esterified fatty acids from WAT, are metabolised in the liver to maintain energy balance. However, dysregulation of these processes, caused by obesity and insulin resistance, leads to health problems such as metabolic dysfunction-associated steatotic liver disease (MASLD) and metabolic dysfunction-associated steatohepatitis (MASH) [130]. Nonalcoholic steatohepatitis (NASH), which leads to liver fibrosis, is a dysfunction of lipid metabolism influenced by the gut–liver axis and the adipose tissue–liver axis [131]. Adipose tissue and the liver play central roles in energy metabolism, nutrient uptake, processing, transport, and storage [132]. Liver and BAT control glycolipid metabolism, thermogenesis, and energy homeostasis. Natural bioactive compounds such as berberine modulate liver and BAT crosstalk via AMPK activation, PPARγ signalling, and UCP1-mediated thermogenesis [133]. Dietary habits play a critical role in controlling obesity; notably, the therapeutic efficacy of ω-3 and ω-6 fatty acids is maximized within a low-carbohydrate, high-lipid dietary protocol [134]. Furthermore, phytochemicals represent key components responsible for sensory properties such as taste, aroma, color, and flavor [135].

## 4. Phytochemicals with Metabolic Reprogramming Properties

**Figure 2:**
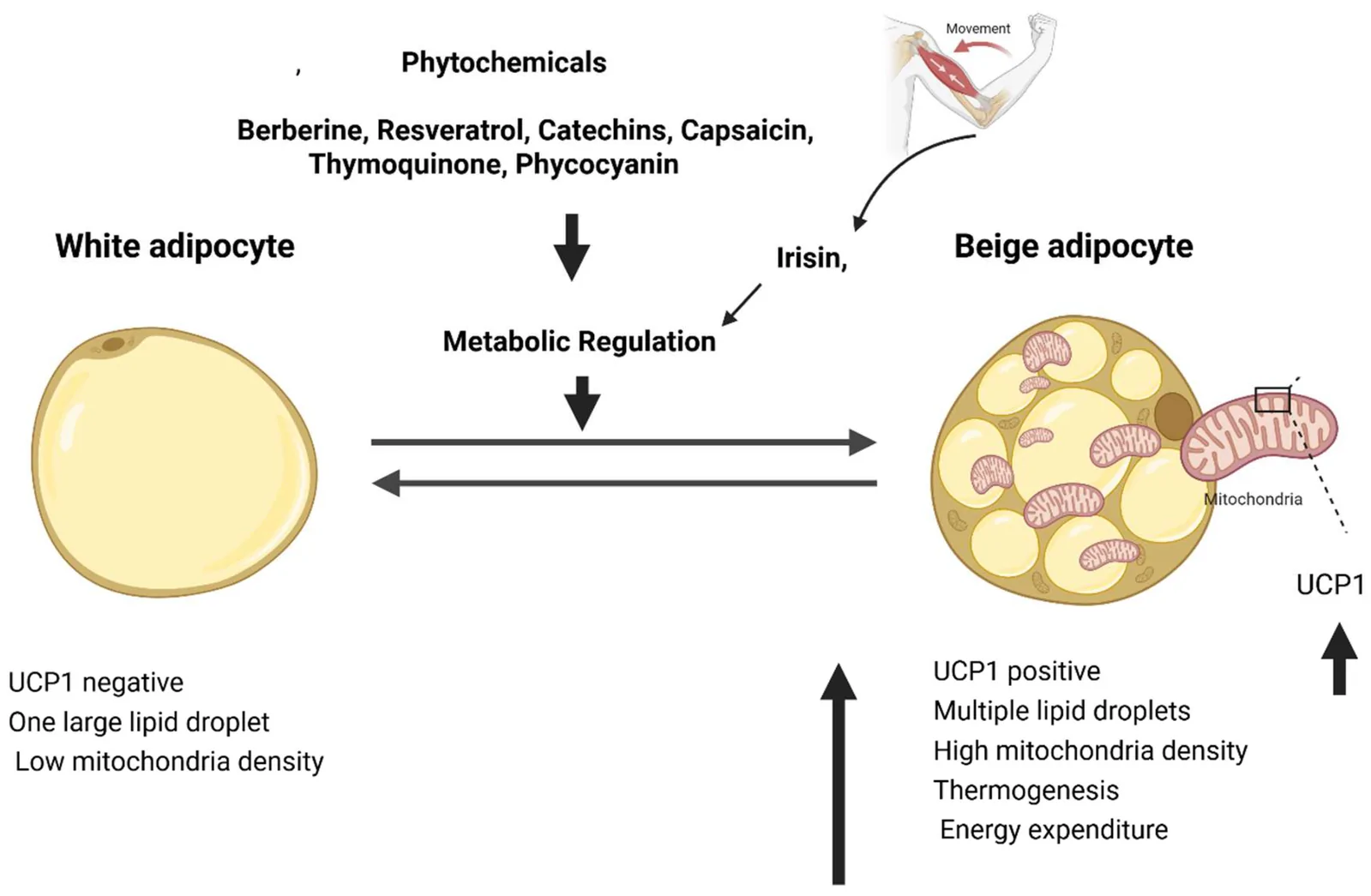
Phytochemical- and irisin-mediated browning of white adipocytes. Bioactive phytochemicals and irisin drive the metabolic transition from white to beige adipocytes, upregulating UCP1 to enhance thermogenesis and energy expenditure.

Several phytochemicals derived from various plant sources have multi-level effects on cellular metabolism in health and disease [136]. They may inhibit glucose absorption from the intestine, suppress the activity of glucose transporters, and inhibit lipid synthesis, but induce lipid catabolism and metabolic energy usage as thermogenesis to lose metabolic energy [137,138]. These compounds induce mitochondrial biogenesis, induce adipose tissue browning, and suppress endoplasmic stress. They generally exhibit minimal adverse effects while promoting health [137,139]. Bioactive phytochemicals and exercise-induced irisin induce the phenotypic transition of adipocytes, resulting in increased mitochondrial density and UCP1-mediated thermogenesis (Figure 2).

### 4.1. Berberine: Mechanisms of Lipolysis and Metabolic Homeostasis

**Figure 3:**
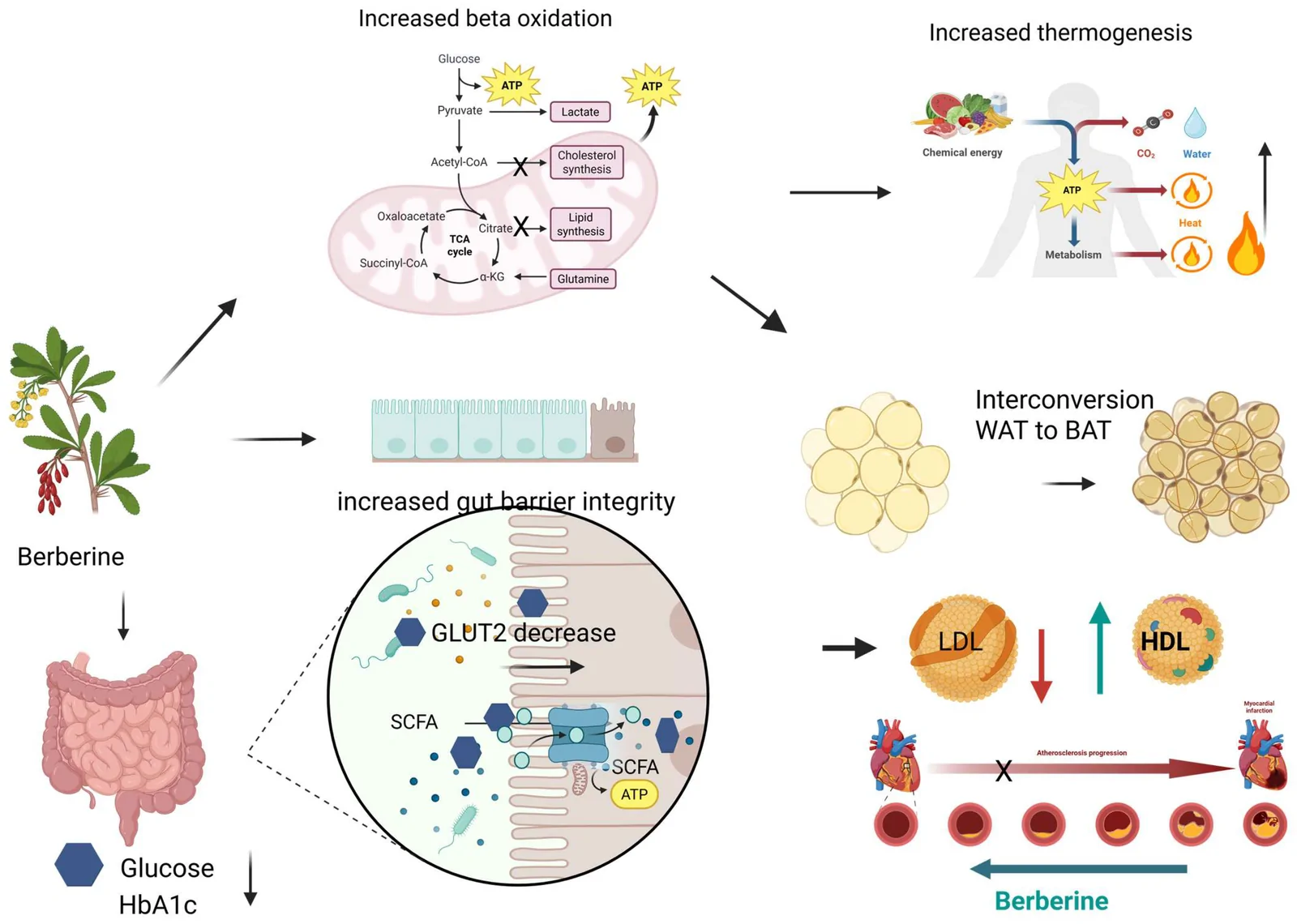
Metabolic effects of berberine. Berberine improves metabolic homeostasis by enhancing gut barrier integrity, modulating gut microbiota, increasing SCFA production, and reducing glucose absorption. It promotes β-oxidation, increases UCP1 expression, enhances thermogenesis, and induces WAT-to-BAT conversion, while improving lipid profiles by decreasing LDL and increasing HDL levels, thereby reducing obesity and metabolic cardiovascular risk.

Berberine is isolated from Coptis chinensis [140] and Berberis vulgaris, as well as from approximately 500 different species worldwide [141]. Berberine is a quinoline alkaloid and has a wide range of pharmacological effects [142]. Berberine has antioxidant, anti-inflammatory, neuroprotective, and cardiovascular protective effects in humans [143]. In various clinical trials, it has been shown to regulate glucose and lipid metabolism, demonstrating significant LDL-lowering, HDL-improving, and insulin-sensitizing effects [143,144]. Berberine activates the signaling pathways of vascular smooth muscle cells, which may make it a promising drug for atherosclerosis [145]. This molecule affects multiple signaling pathways, including AMPK, mTOR, SIRT1/Forkhead box O, FOXO3, Nrf2, NADPH quinone oxidoreductase 1 (NQO1), glutathione peroxidase, and phosphatidylinositol-4,5-bisphosphate-3-kinase catalytic subunit alpha [146]. Berberine induces lipolysis, β-oxidation, and inhibits adipogenesis [26]. It also activates liver kinase (LK)B1, AMPK, and TORC2 [147,148] and inhibits gluconeogenesis by inhibiting the control enzymes of the pathway [149]. It also affects the localization of GLUT2 and decreases glucose absorption from the intestines [150]. Berberine has anti-diabetic, anti-obesity, anti-inflammatory, antibacterial, and geroprotective properties. It has beneficial effects on neurodegenerative diseases. Berberine increases the growth differentiation factor 15 (GDF15), which reduces food intake and appetite, and lowers body weight [140]. The GDF-15 signaling pathway has minimal effect on skeletal muscle mass [151]. Berberine increases insulin sensitivity and regulates gut microbiota [152], decreases blood glucose levels, HbA1c and glycemic index [153]. These beneficial gastrointestinal effects increase SCFAs in the stool, thereby repairing intestinal integrity and reducing inflammation [153,154]. However, berberine has poor bioavailability and intestinal absorption. Berberine is metabolized by phase I and phase II biotransformation, primarily by hepatic cytochrome P450 enzymes [26]. This molecule has adverse effects on the gastrointestinal system when used with metformin or acarbose [155]. The metabolic effects of berberine, including increased β-oxidation, thermogenesis, improved gut barrier integrity, and modulation of lipid metabolism, are illustrated in Figure 3.

Berberine may cause diarrhea or constipation [156], abdominal pain, anorexia, nausea, and flatulence. Berberine has mild gastrointestinal side effects [157]. Transient gastrointestinal symptoms, including diarrhea and constipation, represent the primary dose-limiting side effects of berberine, largely driven by its potent antimicrobial activity against the gut microbiota and its regulatory effects on intestinal motility [158].

### 4.2. Catechins: Antioxidant Activity and Lipid Modulation

The different processing methods of *Camellia sinensis* produce different forms of tea, like black tea, green tea, yellow tea, Matcha, purple tea, fuzhuan tea, oolong, Pu-erh, albino, and white teas [20]. The primary bioactive compounds are catechins, a class of natural polyphenolic phytochemicals. These include EGCG, epigallocatechin (EGC), epicatechin (EC), gallocatechin gallate (GCG), epicatechin gallate (ECG), gallocatechin (GC), and catechin [159,160]. Among the various bioactive compounds identified in *Camellia sinensis*, catechins have the highest antioxidant activity [161].

One of the best-known anti-obesity phytochemicals is catechins and found in several types of tea. They are present in large amounts of green tea and have been associated with weight loss [162]. Green tea catechins (GTC) have beneficial effects on fat metabolism and can modulate energy expenditure, as shown by two studies showing an increase in resting metabolic rate: one reported a modest rise of approximately 183 kJ/day (~44 kcal/day), while another observed an increase of 1091 kJ/day (~261 kcal/day), mainly when subjects participated in resistance training exercise [163].

EGCG alters ubiquitin-proteasome activity, glycolysis, and increases the intracellular ATP level [164]. In human Sertoli cells, a 50 µM EGCG concentration reduced glucose and pyruvate consumption and decreased pyruvate-to-alanine conversion, while maintaining TCA cycle activity [165].

#### 4.2.1. Regulation of Energy Expenditure and Lipotoxicity

Green tea affects metabolism, increases energy expenditure and fat oxidation; on the other hand, it decreases nutrient absorption and appetite [162]. EGCG activates the AMPK pathway, which plays a crucial role in energy homeostasis [166]. AMPK is a heterotrimeric complex, with the catalytic part α and the regulatory subunits β and γ. This complex is activated via Thr-172 phosphorylation triggered by energy stress, hypoxia, upstream kinases (LKB1, CaMKK, and c-Src), and hormones (adiponectin and leptin). The antidiabetic drug metformin promotes this activation by targeting the AMPK pathway [167]. Wang et al. reported that EC inhibits lipid accumulation, inflammation, apoptosis in hepatocytes, decreases oxidative stress, and ameliorates metabolic dysfunction-associated steatohepatitis [168]. Dietary supplementation of different phytochemical combinations (p-synephrine, catechin, or anthocyanins) increased fatty acid oxidation [169]. EGCG attenuates high-fat diet-induced metabolic dysfunction associated with steatotic liver disease (MASLD) by modulating the Nrf2/HO-1 antioxidant defense system and suppressing NF-κB-dependent transcription of inflammatory cytokines IL-6, TNF-α, and MCP-1, and is a bioactive dietary compound [170]. Acid-heated catechins inhibit fatty acid synthetase (FAS) complex in a H+-dependent mechanism distinct from the known FAS inhibitors [171]. EGCG and taurine synergistically regulate lipid metabolism by inhibiting lipogenic enzyme activities [172]. Palmitoyl-epigallocatechin gallate (PEGCG), a lipophilic derivative of EGCG, can reduce proinflammatory cytokines (IL-6, IL-17, IL-18, IL-23, and TNF-α), but PEGCG can more effectively attenuate IL-1β and IL-12p70 due to its fatty acid moiety [173]. Pro-inflammatory cytokines are significantly elevated during obesity-related inflammation [174]. IL-1β, IL-6, and TNF-α are key mediators of obesity-associated low-grade inflammation. [173].

#### 4.2.2. Modulation of Adipocyte Differentiation and Phenotypic Browning

**Figure 4:**
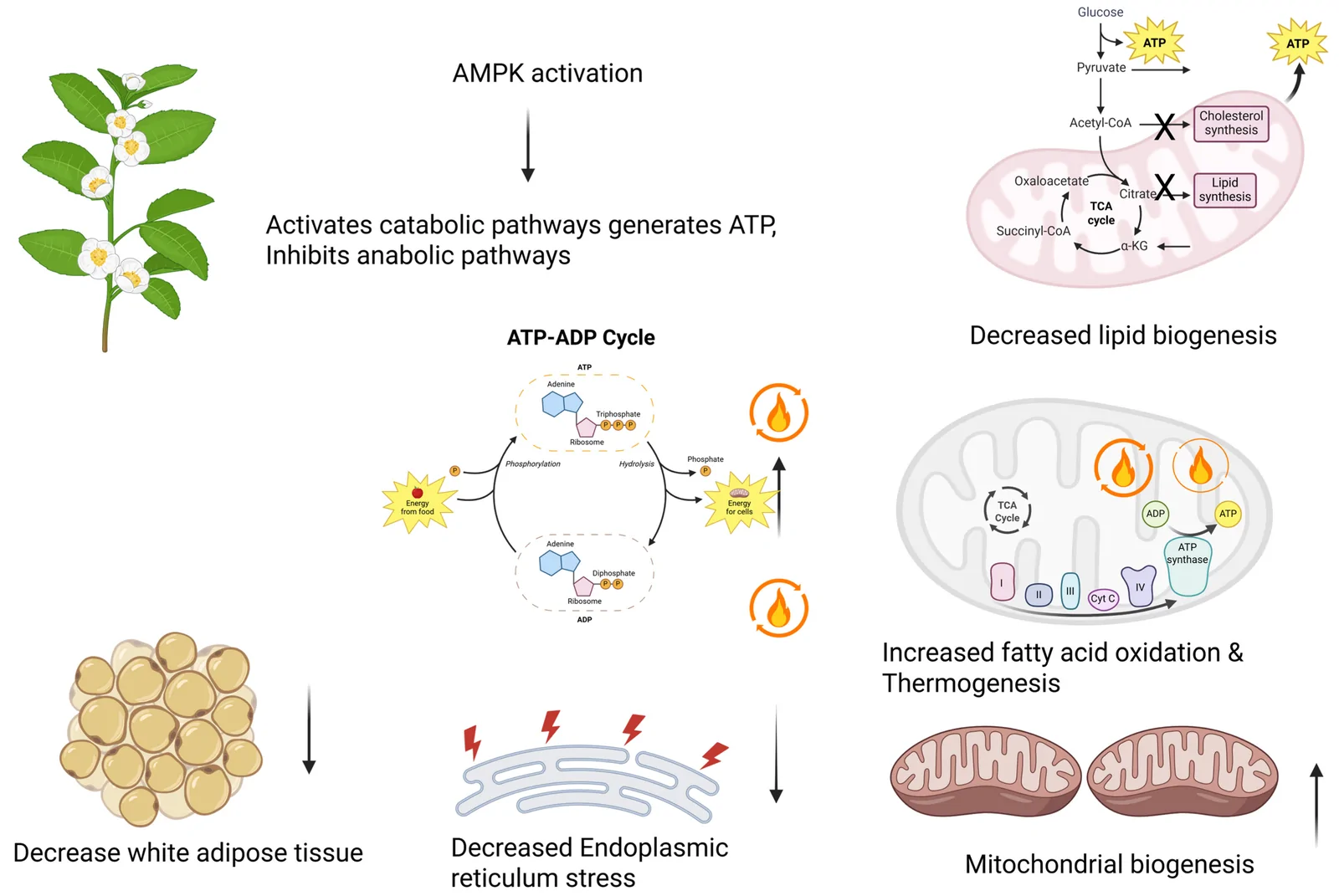
AMPK-mediated regulation of energy metabolism. AMPK activation increases fatty acid oxidation, thermogenesis, and mitochondrial biogenesis while inhibiting lipid synthesis and reducing endoplasmic reticulum stress.

EGC inhibited adipogenesis in a dose-dependent manner and increased adiponectin and UCP1 transcription in mature adipocytes [175]. Cocoa tea (Camellia ptilophylla) is a naturally decaffeinated tea plant with the major catechin EGCG. It has been shown to downregulate adipogenic transcription factors (PPARγ and C/EBPα), adipocyte-specific genes, and MAPK signaling pathways, thereby exerting beneficial effects on obesity, hepatic steatosis, and hyperlipidemia [176]. Catechin promotes adipocyte differentiation and insulin sensitivity in human bone marrow-derived mesenchymal stem cells through dose-dependent activation of PPARγ. This mechanism may contribute to the antidiabetic effects of green tea [177]. Catechin and caffeine treatment inhibited glycerol-3-phosphate dehydrogenase (GPDH)activity and lipid accumulation, but increased heat production and differentiated 3T3-L-1 adipocytes from white to beige [178]. Ulmus davidiana var japonica (UDE) contains catechin as a major active component that inhibits adipogenesis and adipocyte differentiation, reduces body weight and adipose tissue mass, lowers total triglyceride and cholesterol levels and hepatic lipid accumulation, and shows anti-obesity properties [179]. RNA sequencing was utilized to analyze the effects of EGCG on WAT and BAT. Stage- and tissue-specific differentiation patterns were observed in both tissue types [180]. Fang et al. found beneficial effects of tea flavins on gut microbiota, inflammation, AMPK-mediated metabolism, and obesity [181]. Figure 4 summarizes the metabolic pathways regulated through AMPK activation, including enhanced fatty acid oxidation, thermogenesis, mitochondrial biogenesis, and reduced lipid synthesis.

Epicatechins have been shown to reduce oxidative and ER stress, modulate mitochondrial biogenesis, regulate gastrointestinal tract function, and inhibit NADPH oxidase and redox-sensitive signals (NF-κB, JNK1/2) that inhibit the insulin pathway [182]. EGCG inhibits the 5-lipoxygenase enzyme, whose products, the leukotrienes, cause adipose tissue inflammation and related pathologies in obesity [183,184].

EGCG has strong therapeutic potential; however, excessive doses of catechins may cause adverse effects, such as liver injury or hepatitis [185], gastrointestinal irritation, and hepatotoxicity [186].

### 4.3. Capsaicin: Trpv1 Activation and Thermogenic Induction

Capsaicin is the principal bioactive compound in red chili peppers and exerts a range of beneficial health effects on obesity and energy metabolism [187]. Capsaicin specifically acts on the vanilloid 1 (TRPV1) receptor, exerts hypolipidemic and anti-obesity effects, inhibits appetite, enhances energy expenditure, regulates lipid metabolism, and reduces fat accumulation [188,189]. Capsaicin and dihydrocapsaicin reduce endothelial inflammation while promoting nitric oxide synthesis and elevating cellular antioxidant capacity [190]. Capsaicin induces the browning of WAT and increases UCP1 expression, thus directly stimulating mitochondrial biogenesis and promoting energy expenditure [191]. This molecule promotes SIRT1 expression and activity via a TRPV1 channel-dependent increase in intracellular Ca 2+, thus triggering the phosphorylation of Ca 2+/calmodulin activated protein kinase II and AMP-activated kinase. The SIRT1-dependent deacetylation of PPARγ and the transcription factor PRDM-16 facilitates the PPARγ-PRDM-16 interaction to induce browning of WAT [192]. The activation of TRPV1 channels by capsaicin is a critical pathway to prevent obesity [192,193,194]. Bargut et al. indicated that browning of WAT has therapeutic potential against a range of diseases [195]. Leptin and ghrelin have key hormonal properties in the regulation of energy homeostasis. Leptin suppresses appetite and promotes energy expenditure, but ghrelin stimulates hunger and food intake. Dysregulation of leptin signaling elevates ghrelin activity, and capsaicin restores appetite control by modulating these pathways [196]. Dietary intake of capsaicin and its analog capsinoids has been shown to stimulate energy expenditure and enhance fat oxidation [197]. Capsaicin regulates thermogenic and lipid metabolism-associated pathways in WAT, induces apoptosis, and inhibits adipogenesis [198]. Capsaicin exerts anti-obesity effects, modulates gut microbiota, reduces lipopolysaccharide-producing bacteria, attenuates TLR4-mediated chronic low-grade inflammation, restores intestinal barrier function, and upregulates tight junction proteins zonula occludens-1 (ZO-1) and occludin [199]. Capsaicin upregulates PPARγ, UCP-1, and PGC-1α, which promote thermogenesis, suppress appetite, improve insulin sensitivity, and increase glucagon-like peptide-1 (GLP-1) secretion in BAT [200]. Hot pepper juice can modulate appetite [201]. Capsaicin activates AMPK and inhibits the AKT/mTOR pathway, which are major regulators of lipogenesis, and is therefore widely accepted as an anti-lipogenic compound [202]. Capsaicin stimulates glucose uptake and consumption, which is suggested to increase ATP production via the elevation of intracellular Ca 2+ levels [203].

Capsinoids can bind to pain TRPV receptors, change metabolism, and affect pain sensation [124]. However, their consumption may also cause gastrointestinal cramps, stomach pain, nausea, diarrhea, and vomiting [204].

### 4.4. Thymoquinone: Anti-Adipogenic and Antioxidant Properties

Thymoquinone has multifaceted therapeutic potential with pharmacological properties, including neuroprotective, spasmolytic, bronchodilatory, hepatoprotective, renoprotective, gastroprotective, and antioxidant activities [205]. Thymoquinone, the principal bioactive compound found in black cumin seed (*Nigella sativa*), is an anti-adipogenic molecule that suppresses lipogenesis and affects obesity [206]. The thymoquinone molecule affects several adipogenic transcription factors, reduces the expression of C/EBPβ, then C/EBPα and PPARγ, and the activity of GPDH. GPDH enzyme catalyzes the conversion of dihydroxyacetone phosphate to glycerol 3-phosphate, a key step in lipid metabolism. The GPDH enzyme forms glycerol 3-phosphate, which is essential for the formation of the triacylglycerides’ backbone structure [207]. Thymoquinone has protective properties against cardiovascular disease, anti-cancer, anti-diabetic, antioxidant, and beneficial effects on the immune system [208]. Thymoquinone enhances Nrf2 expression, HO-1, superoxide dismutase, and catalase enzyme activities, modulates oxidative stress and inflammation, and controls obesity in a high-fat diet [209].

Thymoquinone has many beneficial effects on human health. It can induce apoptosis and cell cycle arrest in glioblastoma [210], hepatocarcinoma [211], and toxicity depends on the dose [212]. But in only one case, it is reported that a 46-year-old woman had eczema of the scalp, and the causative allergen was Nigella sativa oil contact dermatitis with cross-reactivity to tert-butylhydroquinone [213].

### 4.5. Phycocyanin: Microalgal Defense Against Glucotoxicity

Spirulina is a microalga that contains high amounts of phycocyanin and exhibits antioxidant, anti-inflammatory, immune system-stimulating, hypolipidemic, hypoglycemic, and antihypertensive properties, as well as beneficial effects on intestinal microbiota [214]. Spirulina has antioxidant and anti-inflammatory properties and preserves intestinal epithelial barriers in humans [84]. Spirulina suppresses biomolecular degradation, including lipid peroxidation and DNA damage, and upregulates the enzymatic activities of endogenous superoxide dismutase and catalase [215]. Phycocyanin inhibits adipogenesis, promotes glycogen synthesis, and activates Akt and AMPK signaling pathways in insulin-resistant hepatocytes [216]. Spirulina has anti-obesity properties that inhibit adipogenesis and lipogenesis and downregulate key adipogenic and lipogenic proteins, for instance, C/EBPα, PPARγ, aP2, SREBP1, ACC, and FAS. Spirulina extracts promote adipose tissue browning and thermogenesis. They can induce AMPKα-mediated upregulation of PRDM16, PGC1α, and UCP1, induce mitochondrial biogenesis, and increase energy expenditure [217]. Phycocyanin activates the AKT/mTOR, IRS-1/AKT, and Nrf2 signaling, decreases oxidative stress, increases GLUT4 expression, ATP synthesis, and insulin signaling [218]. C-phycocyanin is a natural pigment in the food industry and has protective effects against high-fat diet-induced metabolic inflammation, reduction in leptin and resistin levels, and decreased NF-κB p50 expression in adipose tissue [219].

**Figure 5:**
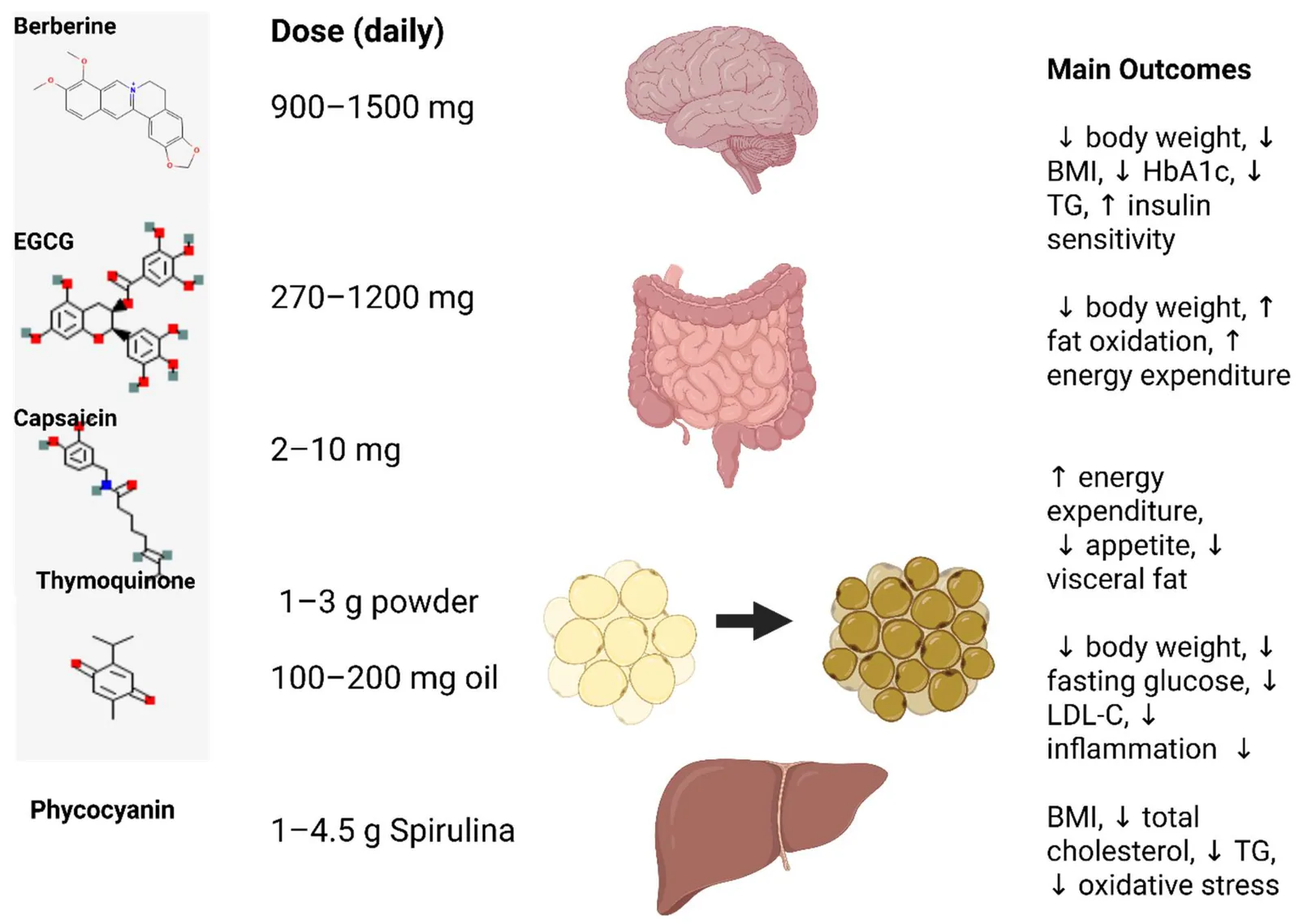
Regulatory effects of bioactive compounds (Berberine, EGCG, Capsaicin, Thymoquinone, Phycocyanobilin) on the brain, gut, adipose tissue (browning), and liver metabolism. Berberine [98,101,102,111], EGCG [120,121,139]; Capsaicin [146,147,149,154,155]; Thymoquinone [165,167,168], Spirulina [173,174,175,177,178].

Phycocyanin has adverse effects on human metabolism, and in one case, it may be an allergen to someone and may cause anaphylaxis [220] (Figure 5).

### 4.6. Resveratrol: Calorie Restriction Mimicry and Tissue Browning

Resveratrol, a polyphenolic compound, has antioxidant, anti-inflammatory, and vasoprotective effects that are beneficial for fatty liver disease, type 2 diabetes, and obesity [28,221,222,223]. This molecule has beneficial effects on WAT browning and induces diverse molecular pathways such as AMPK, PPARγ, SIRT, TRP channels, β3-adrenergic, and FGF21-related signaling cascades that regulate WAT browning [224]. Resveratrol affects mitochondrial function and mimics the effects of calorie restriction [225], but increasing longevity remains unclear [226]. Resveratrol can regulate gut microbiota [227]. The effects of resveratrol supplementation on body composition, adiponectin, and leptin levels in overweight and obese individuals are still uncertain and require further investigation [228]. Resveratrol decreases hepatic steatosis, affects collagen deposition, and normalizes AST, ALT, and ALK enzyme activities [229] (Table 1).

### 4.7. Human Studies on Phytochemicals

According to recent systematic reviews and meta-analyses, berberine supplementation significantly reduces body weight, BMI, waist circumference, and C-reactive protein (CRP) levels, without exerting adverse effects on liver enzymes (ALT and AST) [230], while exerting well-documented beneficial effects on insulin resistance and glycemic control in humans [231]. Current human clinical trials demonstrate that oral curcumin supplementation yields significant therapeutic benefits, particularly in inflammation-driven metabolic and musculoskeletal disorders [232]. Co-administration of curcumin with piperine overcomes its low oral bioavailability, thereby modulating metabolic pathways related to glycemic control, lipid profiles, and inflammatory responses in human clinical conditions such as diabetes and metabolic syndrome [233]. In overweight and obese patients, resveratrol administration effectively enhances the overall metabolic profile through its anti-inflammatory, antioxidant, and anti-lipidemic properties [234]. Human and pre-clinical studies demonstrate that green tea and its primary catechin, EGCG, attenuate obesity-related inflammatory diseases by scavenging reactive oxygen species and downregulating NF-κB-mediated cytokine expression [235]. EGCG exhibits potent anti-obesity and anti-inflammatory properties; its clinical application in humans is hindered by poor oral bioavailability and low stability, limitations that can be effectively overcome through nanoparticle encapsulation strategies to optimize its therapeutic delivery [236]. Targeting the global obesity pandemic, dietary capsaicin consumption provides an accessible therapeutic approach to body weight reduction through its systemic regulation of metabolic health and tissue-level energy expenditure [237]. Finally, clinical evidence confirms that thymoquinone, the primary bioactive compound of *Nigella sativa*, significantly improves metabolic profiles in obese individuals by reducing body weight, fasting blood glucose, and systemic inflammatory markers [238].

### 4.8. How Selected Phytochemicals Interact with the GLP-1/GLP-1R Axis

Natural products like berberine, EGCG, resveratrol, and curcumin are well known to modulate GLP-1 expression and secretion [239]. For example, capsaicin triggers acute GLP-1 release from L-cells via transient receptor potential vanilloid 1 (TRPV1) activation [240].

Berberine metabolites stimulate GLP-1 secretion by fighting oxidative stress and mitochondrial dysfunction [241]. Thymoquinone stimulates GLP-1 release in diabetic models by selectively targeting and activating intestinal imidazoline receptors [242]. Peptides derived from *Spirulina* phycocyanin show strong in vivo antihyperglycemic effects [243]. This is largely because their DPP-IV inhibitory activity protects endogenous GLP-1 from rapid degradation [244].

These phytochemicals do more than increase GLP-1 levels. They can amplify the cellular signals triggered by activation of the GLP-1 receptor. They also affect other metabolic pathways; berberine suppresses hepatic gluconeogenesis primarily through the LKB1-AMPK-TORC2 pathway [245,246]. Thymoquinone exerts its anti-neuroinflammatory effects in microglia through the sequential activation of the AMPK and NAD+/SIRT1 signaling pathways [247]. EGCG and resveratrol increase skeletal muscle mitochondrial capacity and sustain systemic fat oxidation. At the cellular level, these polyphenols directly reinforce GLP-1R downstream signaling by inhibiting phosphodiesterases and elevating intracellular cAMP levels [248].

### 4.9. Selection Criteria for Phytochemicals:

The bioactive phytochemicals in this review were selected based on three specific criteria: (1) proven capacity to induce white adipose tissue browning and UCP1 expression, (2) demonstrated ability to modulate key metabolic signaling pathways (such as AMPK, SIRT1, and GLP-1/irisin axes), and (3) availability of well-documented human clinical or translational data in obesity management.

## 5. Glucagon-like Peptide-1 Receptor Agonists and Multi-Receptor Co-Agonists

### 5.1. Semaglutide: Single Glp-1r Agonism and Pharmacokinetic Optimizations

Semaglutide is a synthetic pharmacological molecule accepted as a GLP-1 receptor agonist [249]. This molecule is an antidiabetic drug that induces adipose tissue browning via increasing UCP-1 and SIRT-1-dependent thermogenic pathways [250]. Semaglutide has three brand names: Ozempic^®^ (Novo Nordisk, Singapore), Wegovy^®^ (Novo Nordisk, Singapore) as injectables, and Rybelsus^®^ (Novo Nordisk, Singapore) for oral intake [251]. Its mechanisms involve glucose, lipid, and organic acid metabolism [252], activating AMPK/SIRT1/NF-κB signaling [253]. It has been reported that semaglutide positively affects glucose tolerance and insulin resistance [254] via activation of SIRT1 expression [255,256]. Semaglutide affects the brain–gut–adipose tissue axis, suppresses appetite, and has anti-inflammatory effects [164], adipocyte browning, mitochondrial biogenesis [165], and antioxidant effects [254]. Semaglutide has beneficial effects on major cardiovascular risk factors among overweight individuals [249] and steatohepatitis [257]. Specifically inhibits squamous cell carcinoma growth by activating the p38 MAPK signaling pathway without affecting ERK1/2 or SAPK/JNK [258].

The secret of semaglutide is its pharmacokinetic properties because this molecule’s half-life is longer than that of a natural GLP-1 agonist [259]. Liraglutide, semaglutide, dulaglutide, and albiglutide are all GLP-1 receptor agonist and, based on their structural or delivery modifications, including N- and C-terminal modifications, they are stable and protect their bioactivity against degradation by dipeptidyl peptidase-IV (DPP-IV) [260]. The half-life of this molecule increased to 160 h by various structural modifications. These include the eighth position, where alanine is replaced with α-aminobutyric acid to protect from degradation by the DPP-4 enzyme. A C18 fatty acid attached to the 26th position of lysine provides binding to albumin, while the 34th position of lysine is replaced with arginine to prevent the incorrect binding of the fatty acid [261].

### 5.2. Tirzepatide: Dual Gip/Glp-1 Receptor Co-Agonism

Tirzepatide is the first dual GIP/GLP-1 receptor co-agonist for the treatment of type 2 diabetes, obesity, and related comorbidities [262] and, in an obese patient with heart failure, reduces weight and lowers blood pressure [263]. Tirzepatide activates the AMPK/NF-κB signaling pathway and has anti-inflammatory and metabolic-regulating properties [264]. Gojani et al. investigated that combining 1 mM metformin with 10 nM semaglutide or 10 nM tirzepatide provides superior protection for INS-1 β cell survival under high-glucose, high-lipid-induced apoptosis and cell cycle dysregulation [265]. Tirzepatide experimental results showed that this drug reduces appetite, body weight, adipose tissue weight, and abdominal fat volume [266]. Tirzepatide increases insulin sensitivity by reducing systemic levels of insulin resistance-associated branched-chain amino acids and keto acids, a metabolic improvement that is coupled with the upregulation of their catabolic genes within thermogenic brown adipose tissue [267]. Therefore, combining novel molecules under a drug may offer potential synergistic metabolic benefits (requires further validation).

### 5.3. Retatrutide: Triple Gip/Glp-1/Glucagon Receptor Tri-Agonism

Retatrutide is one of the latest weight loss medications for obesity [268]. This is a triple agonist targeting GLP-1, GIP, and glucagon receptors [269]. Classified as a multi-receptor therapeutic [270] and enhances insulin secretion, optimizes glucose homeostasis, and improves appetite modulation [271]. This drug decreases the respiratory exchange ratio and increases BAT UCP1 expression [272] and has many metabolic health benefits [273]. Retatrutide transforms WAT into a metabolically important organ, increasing lipid oxidation, decreasing inflammation, and restoring endocrine function [274]. This drug has beneficial effects on metabolic dysfunction and on reducing hepatic and cardiovascular risk in type 2 diabetes and in metabolic dysfunction-associated steatotic liver disease therapy [275].

All GLP-1 receptor agonists may have adverse effects on the pancreas and gallbladder, such as pancreatitis and gallbladder disease [276], as well as other gastrointestinal symptoms, including nausea, diarrhea, constipation, and abdominal discomfort [277].

## 6. Crosstalk Between Glp-1 Receptor Agonists and Bioactive Phytochemicals at the Ampk/sirt1 Signalling

### 6.1. Berberine as a Natural Glp-1 Enhancer and Epigenetic Regulator

Glucagon-like peptide-1 (GLP-1) is primarily synthesized in two locations in humans: firstly, by the enteroendocrine L-cells of the distal ileum and colon in response to nutrient intake, and secondly, in the nucleus tractus solitarius (NTS) of the brainstem [278,279]. The pharmacological drugs semaglutide and liraglutide are true, direct agonists that bind specifically to the GLP-1 receptor [280]. Phytochemicals affect metabolism in diverse ways [281]. Berberine, quercetin, curcumin, EGCG, flavonoids, terpenoids, alkaloids, and polyphenols operate as natural GLP-1 enhancers [282,283] or secretagogues that stimulate endogenous GLP-1 secretion from intestinal L-cells or potentially decrease its enzymatic degradation [284].

GLP-1 functions as a regulator of appetite and energy homeostasis in the gut–brain axis [279]. GLP-1 is a major metabolic hormone that regulates insulin secretion by suppressing glucagon secretion [285] and exerts broad pharmacological effects [278]. GLP-1 receptor agonists (GLP-1 RAs) play important roles in managing obesity and type 2 diabetes, lowering HbA1c, and regulating metabolism [286]. GLP1-RAs are a group of antidiabetic drugs that restore glucose homeostasis, suppress appetite, and enhance satiety and gastric emptying through the activation of GLP-1 receptor signaling [287]. These drugs also have anti-inflammatory, anti-thrombotic, anti-obesogenic, and beneficial cardiovascular and renal properties [288]. GLP1-RAs are analogs of incretins. These agonists bind their G protein-coupled receptor, which triggers and activates adenyl cyclase and the release of cAMP [289]. Activation of cAMP signaling leads to PKA activation, which phosphorylates CREB and indirectly regulates AMPK signaling, thereby modulating ferroptosis and fatty acid synthesis [290].

In the GLP-1 signaling pathway, cAMP can activate Rap1 via EPAC (Exchange Protein directly activated by cAMP) and the PI3K/Akt pathway, plus inhibit gluconeogenic enzymes [291]. GLP-1 receptor signaling pathway, the MAPK/extracellular signal-regulated kinases 1/2 pathways also activate and regulate cell proliferation, differentiation, and transformation [292]. GLP-1 receptor agonists increase β-cell sensitivity to glucose by modulating K_ATP_ channel activity and intracellular calcium concentration, which enhances insulin secretion [293].

These phytochemicals (berberine, resveratrol, catechins, capsaicin, thymoquinone, and phycocyanin) modulate key components of classical energy-regulating signaling networks, including adenylate cyclase, cAMP, PKA, CREB, and AMPK pathways, through both direct and indirect mechanisms [294,295,296,297,298,299]. Berberine and its metabolites are multi-target therapeutic agents with important pharmaceutical effects in the management of obesity [26]. Berberine may enhance endogenous GLP-1 secretion, trigger the G protein-coupled receptor complex, and activate the signaling pathway, which is directly related to energy homeostasis [300] and to the activation of lipolysis and β-oxidation. PPAR-γ inhibits adipogenesis, affects the gut microbiota [301] and UCP1, and promotes adipose tissue browning [26], thereby contributing to the management of type 2 diabetes [302]. Berberine is one of the phytotherapeutic alternatives with GLP-1-enhancing properties. Berberine may inhibit skeletal myofibrillogenesis; unlike UPS inhibitors or semaglutide, it exerts no adverse effects on cardiac muscle assembly [303]. Berberine integrates insulin signaling, SIRT1/3-mediated mitochondrial adaptation, fatty acid synthesis, ferroptosis, NF-κB, and NLRP3 inflammatory pathways in managing obesity [304]. Berberine inhibits DNA methyltransferase 1 (DNMT1) and histone deacetylase 3 (HDAC3), progressive silencing may modulate insulin signaling gene expression via epigenetic regulation [305], and p53-dependent apoptosis and DNA methyltransferase 3b (DNMT3b) [306]. Inhibition of DNA methylation and histone acetylation enzymes by berberine shows that berberine also affects lipid metabolic disease and anti-obesity via epigenetic modulation [307]. Berberine has beneficial effects on high-fat diet-induced NAFLD [308]. Berberine has drug-like pharmacological properties [309]. Metformin and berberine have similar effects on metabolism and NAFLD. These molecules activate the AMPK signaling pathway, which suppresses SREBP1/FASN to inhibit de novo lipogenesis [310]. Berberine primarily regulates metabolic pathway activity by mimicking the effects of GLP-1 receptor agonists and metformin [287]. In contrast, resveratrol acts as a potent caloric restriction mimetic, with both compounds converging at the AMPK/SIRT1 node to orchestrate metabolic reprogramming [311].

### 6.2. Synergy of Glp-1 Receptor Agonists and Resveratrol: Shared Ampk/sirt1 Signaling

GLP-1 receptor agonists and resveratrol share metabolic regulatory activation of the AMPK pathway [312]. Resveratrol activates AMPK, which in turn activates SIRT1; SIRT1 then regulates insulin sensitivity and glucose metabolism via AMPK/FOXO1 and IRS-1/Akt [313]. GLP-1 receptor agonists mainly act through the cAMP-PKA pathway [314]. Resveratrol and GLP-1 receptor agonist effects ameliorate oxidative stress, inflammation, mitochondrial dysfunction, and apoptosis [315,316]. The overlapping activation of AMPK/SIRT1 signaling suggests that GLP-1 receptor agonists and resveratrol may exert complementary or potentially synergistic metabolic effects, although this hypothesis requires validation in controlled clinical studies [317,318]. GLP-1 receptor agonists regulate lipid metabolism, such as the induction of lipolytic proteins, and induce autophagy via the AMPK/SIRT1 signaling pathway [319]. GLP-1 receptor agonists have inhibited mTOR and glucose-induced mitochondrial damage [320], and the same is true for resveratrol. Resveratrol also protects against mitochondrial damage from high glucose levels [321]. Resveratrol may inhibit inflammatory cytokines, reduce inflammation and oxidative stress, and activate the endogenous antioxidant system by activating Nrf2 [322]. On the other hand, resveratrol treatment, alone or combined with Vitamin D, failed to improve aortic biomechanics or mineral levels. Unlike Vitamin D, it demonstrated no protective effects against T2DM-induced cardiovascular damage [223].

## 7. Conclusions

In conclusion, bioactive phytochemicals such as berberine, resveratrol, catechins, capsaicin, thymoquinone, phycocyanin, and the exercise-induced hormone irisin offer a potent dual approach as antioxidants and metabolic regulators. By modulating AMPK signaling to promote mitochondrial biogenesis and upregulate UCP1, these compounds effectively drive thermogenesis and adipose tissue browning, thereby fortifying the brain–gut–adipose tissue–liver axis. As a result, metabolic reprogramming effectively drives weight loss. Among these diverse metabolic pathways, the induction of WAT browning and UCP1-mediated thermogenesis emerges as the mechanism with the greatest translational and therapeutic potential. By actively increasing energy expenditure rather than relying solely on calorie restriction or appetite suppression, this synergistic axis offers a highly sustainable, physiologically aligned, and non-invasive therapeutic approach to combat obesity and its associated metabolic comorbidities. Importantly, this core thermogenic mechanism is synergistically amplified by secondary systemic pathways described herein. Specifically, phytochemicals optimize the gut microbiota to generate SCFAs that trigger sympathetic browning pathways. Simultaneously, anti-inflammatory remodelling marked by the shift from M1 to M2 macrophages improves tissue inflammation, unlocking the metabolic potential of beige adipocytes. This highlights the clinical value of a systems-level, rather than single-target, anti-obesity strategy. These phytochemicals should not be viewed as isolated bioactive compounds acting through single molecular targets, but rather as network regulators that converge on evolutionarily conserved nutrient-sensing pathways, including AMPK, SIRT1, Nrf2, PPARγ, and mitochondrial signaling. Their ability to simultaneously influence energy metabolism, inflammation, oxidative stress, gut microbiota, and inter-organ communication highlights the importance of systems-level metabolic regulation in obesity management.

Phytochemicals may complement GLP-1 receptor agonists in existing obesity therapies by targeting common metabolic pathways involved in energy homeostasis, inflammation, and mitochondrial function. Future therapeutic strategies for obesity are likely to integrate lifestyle interventions, exercise-induced mediators such as irisin, phytochemical-based metabolic modulators, and GLP-1 receptor agonists to achieve personalized and sustained metabolic health.

## 8. Limitations and Future Directions

While the synergistic integration of irisin and phytochemicals offers a promising therapeutic framework for obesity, translating these pre-clinical findings into clinical practice requires addressing several critical limitations. Additional experimental and clinical data are still needed to establish precise, safe, and effective dosage ranges for both single and combined applications, while carefully evaluating potential drug-phytochemical interactions. Furthermore, utilizing advanced delivery systems, such as liposomes or nanoparticle-based formulations, remains critical to overcome low bioavailability and maximize therapeutic efficacy. Given the systemic complexity of obesity, future research should transition from single-target approaches toward a multi-organ perspective. Specifically, prospective studies must investigate whether recombinant irisin, when combined with these phytochemicals or with existing pharmacological agents such as GLP-1 agonists, can produce additive or synergistic effects on metabolic regulation, insulin sensitivity, and adipose tissue browning, thereby opening new horizons for personalized therapeutic strategies.

Importantly, integrating these multi-target agents demands careful evaluation of potential synergistic contraindications and dose-dependent adverse reactions. Even as isolated compounds, certain phytochemicals exhibit notable baseline toxicities; for instance, berberine frequently induces gastrointestinal distress, while high doses of green tea catechins are clinically associated with hepatotoxicity. When co-administered, these compounds may cooperatively inhibit hepatic cytochrome P450 enzymes, severely impairing the clearance of concurrent medications and exacerbating systemic toxicity. Furthermore, excessive or uncontrolled activation of the irisin-mediated browning pathway risks driving the metabolic rate beyond safe physiological limits, potentially leading to muscle catabolism (sarcopenia) or elevated cardiovascular strain. Consequently, because any alteration in phytochemical combinations or dosage ratios completely resets the safety profile, these formulations must be treated as novel therapeutic candidates. This requires a step-by-step drug development process: starting with cell culture models, moving to multi-lineage organoids to test enzyme toxicity, progressing to animal studies, and ending with Phase I–III human clinical trials to ensure safety and efficacy.

To effectively manage and reduce these clinical challenges, several practical strategies can be used. First, using advanced targeted delivery systems, such as enteric-coated capsules or nanoparticle-based formulations, can shield the stomach from direct mucosal irritation while bypassing first-pass hepatic metabolism, thereby reducing the required systemic dose. Using structural modifications, such as prodrug designs, can also ensure that active agents remain inert until they reach specific target tissues. Second, clinical protocols should adopt a gradual dose-escalation scheme alongside chemical buffering, co-administration of mucosal-protecting agents, and targeted probiotics to maintain gut barrier integrity. Finally, using chronotherapy to stagger the administration times of phytochemicals and concurrent prescription drugs can prevent acute, competitive inhibition. When combined with pre-treatment pharmacogenomic screening to identify individual metabolic rates and routine liver enzyme (ALT/AST) monitoring, these strategies collectively improve clinical safety. Choosing phytochemicals with better pharmacokinetic profiles, specifically those with higher bioavailability and less reliance on liver metabolism, helps lower hepatic strain without losing therapeutic effect. In the same way, sustained-release or colon-targeted formulations can prevent sudden spikes in the gut, making the treatment much easier to tolerate. At the microbiome level, adjusting the patient’s diet or using specific prebiotics and combination therapies of pre- and probiotics can support healthy gut barrier function and prevent local inflammation. To prevent dangerous drug-phytochemical interactions, especially in patients taking multiple medications, a multidisciplinary team of physicians and clinical pharmacists must carefully review and reconcile all active prescriptions. Also, for co-administered drugs with a narrow therapeutic index, clinicians should combine therapeutic drug monitoring with regular checks of liver enzymes and inflammatory biomarkers to catch and address early signs of toxicity. Doses should likewise be flexible, adjusted continuously based on the patient’s age, comorbidities, organ function, and overall tolerability. To do this work, educating patients on simple habits like taking doses with meals, staying hydrated, and recognizing early side effects is key to helping them stay on treatment. Looking ahead, machine learning models and precision medicine tools can help predict patient-specific drug clearance and identify individuals at high risk of liver or gut toxicity. Nevertheless, these strategies require validation through well-designed preclinical studies and randomized clinical trials before they can be recommended for widespread clinical use.

## Figures and Tables

**Table 1:** Summary of the major metabolic effects, clinical evidence, microbiota interactions, and side effects of selected phytochemicals.

Molecule	Molecular Targets	Effects on Adipose Tissue	Effects on Liver Metabolism	Effects on Energy Metabolism	References
**Berberine**	AMPK, SIRT1, PI3K/Akt, mTOR, FOXO3, Nrf2, NQO1, GDF15	Inhibits adipogenesis, promotes lipolysis	Reduces hepatic lipid accumulation, improves insulin sensitivity	Enhances glucose uptake, decreases intestinal glucose absorption, and increases beta-oxidation	[25,26,140,141,142,143,144,145,146,147,148,149,150,151,152,153,154,155,156,157,158]
**Catechins**	AMPK, Nrf2, PPARs (PPARγ, C/EBPα), FAS, NF-κB	Stimulates thermogenesis and fat oxidation, inhibits adiposity and GPDH activity	Reduces hepatic steatosis, attenuates MASLD, and mitigates oxidative stress	Increases energy expenditure	[20,159,160,161,162,163,164,165,166,167,168,169,170,171,172,173,174,175,176,177,178,179,180,181]
**Capsaicin**	TRPV1, AMPK, SIRT1, PRDM-16, PGC-1α, AKT/mTOR	Promotes browning of WAT, induces apoptosis, and suppresses adipogenesis	Improves lipid metabolism and attenuates TLR4-mediated inflammation	Increases thermogenesis, enhances fat oxidation, and stimulates glucose consumption	[187,188,189,190,191,192,193,194,195,196,197,198,199,200,201,202,203,204]
**Thymoquinone**	Nrf2, PPARγ, NF-κB, C/EBPβ, C/EBPα, GPDH	Inhibits adipocyte differentiation and suppresses lipogenesis	Reduces inflammation, decreases oxidative stress, and exerts hepatoprotective effects	Improves overall metabolic balance and homeostatic control	[205,206,207,208,209,210,211,212,213]
**Phycocyanin**	AMPK, AKT/mTOR, IRS-1/AKT, Nrf2, SREBP1, ACC, FAS	Reduces adipocyte lipid accumulation, downregulates lipogenic proteins, and promotes WAT browning	Protects against hepatic oxidative damage and counteracts metabolic inflammation	Inhibits hepatic gluconeogenesis, promotes glycogen synthesis, and enhances ATP production	[214,215,216,217,218,219,220]
**Resveratrol**	AMPK, PPARγ, SIRT1, TRP channel, beta3-adrenergic, and FGF21 pathways	Promotes WAT browning	Decreases hepatic steatosis and normalizes transaminase (AST, ALT, ALK) activities	Mimics the molecular effects of calorie restriction and enhances mitochondrial function	[28,221,222,223,224,225,226,227,228,229]
**Irisin**	PGC-1α, UCP1, AMPK, BDNF	Drives phenotypic transition from white to beige adipocytes, enhancing UCP1-mediated thermogenesis	Suppresses gluconeogenesis and lipogenesis, improving insulin sensitivity	Increases energy expenditure and mimics the metabolic benefits of calorie restriction	[99,100,101,102,103,104,105,106,107,108,109,110,111,112,113,114,115]

## Data Availability

No datasets were generated or analyzed during the current study. All figures were created with BioRender.com.
